# Understanding teacher design practices for digital inquiry–based science learning: the case of Go-Lab

**DOI:** 10.1007/s11423-020-09904-z

**Published:** 2021-01-11

**Authors:** Ton de Jong, Denis Gillet, María Jesús Rodríguez-Triana, Tasos Hovardas, Diana Dikke, Rosa Doran, Olga Dziabenko, Jens Koslowsky, Miikka Korventausta, Effie Law, Margus Pedaste, Evita Tasiopoulou, Gérard Vidal, Zacharias C. Zacharia

**Affiliations:** 1 University of Twente, Enschede, the Netherlands; 2 École Polytechnique Fédérale de Lausanne, Lausanne, Switzerland; 3 Tallinn University, Tallinn, Estonia; 4 University of Cyprus, Nicosia, Cyprus; 5 Information Multimedia Communication AG, Munich, Germany; 6 Núcleo Interactivo de Astronomia, São Domingos de Rana, Portugal; 7 Universidad de La Iglesia de Deusto, Bilbao, Spain; 8 Ellinogermaniki Agogi Scholi Panagea Savva Ae, Pallini, Greece; 9 University of Turku, Turku, Finland; 10 University of Leicester, Leicester, UK; 11 University of Tartu, Tartu, Estonia; 12 EUN Partnership aisbl, Brussels, Belgium; 13 École Normale Supérieure de Lyon, Lyon, France

**Keywords:** Digital education, Online labs, Instructional design process, Learning analytics, Inquiry learning

## Abstract

Designing and implementing online or digital learning material is a demanding task for teachers. This is even more the case when this material is used for more engaged forms of learning, such as inquiry learning. In this article, we give an informed account of Go-Lab, an ecosystem that supports teachers in creating Inquiry Learning Spaces (ILSs). These ILSs are built around STEM–related online laboratories. Within the Go-Lab ecosystem, teachers can combine these online laboratories with multimedia material and learning apps, which are small applications that support learners in their inquiry learning process. The Go-Lab ecosystem offers teachers ready–made structures, such as a standard inquiry cycle, alternative scenarios or complete ILSs that can be used as they are, but it also allows teachers to configure these structures to create personalized ILSs. For this article, we analyzed data on the design process and structure of 2414 ILSs that were (co)created by teachers and that our usage data suggest have been used in classrooms. Our data show that teachers prefer to start their design from empty templates instead of more domain–related elements, that the makeup of the design team (a single teacher, a group of collaborating teachers, or a mix of teachers and project members) influences key design process characteristics such as time spent designing the ILS and number of actions involved, that the characteristics of the resulting ILSs also depend on the type of design team and that ILSs that are openly shared (i.e., published in a public repository) have different characteristics than those that are kept private.

## Introduction

There is a growing need in our society for highly qualified workers in STEM (Science, Technology, Engineering and Mathematics) who not only have deep knowledge of their domain, but who are what are called T–shaped professionals, able to be reflective and to work in teams on complex problems (Sa’ad and Peers 2012). This calls for a change in educational approach, with a strong emphasis on forms of engaged learning in combination with the acquisition of twenty-first century skills. Technology can support teachers in realizing this change, by offering interactive domain–related applications (e.g., online laboratories) that enable active or engaged learning and that provide students with tools that support these engaged forms of learning and tools that train them in twenty-first century skills.

A solution has been developed and implemented that realizes these goals for the STEM field. On its sharing platform (see www.golabz.eu), the Go-Lab ecosystem offers learning resources (Inquiry Learning Spaces, or ILSs in short) that promote inquiry learning and that have online laboratories as their core component. In ILSs, these online labs are enriched with tools (apps) to support the inquiry process, such as apps to help students state hypotheses or design experiments, and with apps to train twenty-first century skills such as collaboration and reflection. Go-Lab also offers an authoring^1^ platform (see www.graasp.eu) for teachers to design their own personalized ILSs. The Go-Lab ecosystem is being used in Europe and worldwide on a very large scale, with more than 37,000 registrations from 152 countries on the authoring platform as of the reference data of December 31, 2019 and more than 440,000 different visitors to the sharing platform. As such, Go-Lab functions as a hub that brings together all types of initiatives worldwide (e.g., it collects a large series of resources from third–party lab repositories, such as PhET, Concord, and Amrita, under one umbrella) and that serves as a bridge between educational providers, in our case often lab owners and the educational field. It also functions as a catalyst for teacher cooperation.

In the current article, we describe the Go-Lab ecosystem from the perspective of the teacher/designer. We highlight the different design and customization options the system offers for creating online or digital inquiry–based learning spaces and explore selected usage data to investigate how these options have been used by teachers in the field. First, however, we present a brief introduction to how Go-Lab got started.

## Engagement in STEM learning: inquiry learning and online laboratories

Engagement in learning is generally seen as one of the driving forces behind acquisition of deep conceptual knowledge. Engagement can be viewed on a more meso level, such as the inclination to come to school on time or being interested in pursuing a job in a specific field (see OECD 2013) or on a more micro level, which is reflected in the cognitive learning processes that students apply. On the learning processes level, engaged learning means that students apply deep cognitive processes such as elaborating, abstracting, and relating that change the information given to them (de Jong 2019). The ICAP framework (Chi 2009; Chi and Wylie 2014) is relevant in this context. The ICAP framework distinguishes several levels of student involvement with the learning material, which range across Passive, for example, watching a video, Active, for example, making links with other material, Constructive, such as creating artifacts like a blog, and Interactive, for example, when discussing with others. Based on other studies and on their own work, Chi and Wylie (2014) stated that interactive learning leads to the best performance, followed by constructive and active learning, with passive approaches being the least effective. Overall, there is consensus in the literature that engagement in learning has a clear relation with completion of (digital) courses (see for example, Soffer and Cohen 2019).

Engaged learning can be prompted by many different forms of instruction, such as problem–based learning, project–based learning, peer tutoring, explanation–based learning, and collaborative learning. For STEM domains, one of the most obvious instructional interventions that stimulates engagement is inquiry learning. The central activity in inquiry learning is to do investigations, which implies the design and performance of experiments based on a research question (see e.g., Bell et al. 2005). In an experiment, students have to manipulate the values of variables and observe the effect of these manipulations on the values of other variables, and in this way infer how the underlying mechanisms work. They can do this to test and possibly adapt their existing knowledge and/or to generate new knowledge and create artefacts such as concept maps or hypotheses, which represents the active and constructive levels of processing from the ICAP framework. Inquiry learning is also very well suited for collaboration, thus also touching upon the interactive level of the ICAP framework.

Traditionally, inquiry learning in STEM topics has centered on performing investigations in hands–on laboratories, but nowadays there is an alternative to this approach in the form of online (virtual and remote or dataset–based) laboratories (see e.g., de Jong et al. 2013). Popular repositories of online labs or simulations include PhET (Moore and Perkins 2018; Wieman et al. 2008), Amrita/OLabs (Achuthan et al. 2011; Nedungadi et al. 2015, 2018), Molecular Workbench/Concord labs (Xie et al. 2011), Physics Aviary (MacIsaac 2015), Physlet Physics (Christian and Belloni 2003), ChemCollective (Yaron et al. 2010), Apps on Physics by Walter Fendt, Galileo and Einstein (Fowler 2009), and many more. The Go-Lab ecosystem (de Jong et al. 2014; Gillet et al. 2013) brings labs from the majority of these collections as well as many smaller collections and single labs together in one portal, in this way offering a one–stop shopping experience; it currently holds over 600 online laboratories.

Online labs only get real instructional value when embedded in an instructional context (de Jong in press). This instructional context can be provided by a teacher who uses an online lab in a class setting, but it can also be offered by online or offline material. A number of the collections mentioned above offer this kind of learning support for online laboratories in the form of additional material, exercises, assignments, tutorials, tests, and so forth. Only a few online lab providers offer interactive apps (scaffolds) for learning (e.g., Inq-ITS; see Gobert et al. 2018). Two online systems offer online labs embedded in online learning resources, interactive scaffolding tools, and authoring facilities for teachers so that they can build their own learning environments that integrate online labs. These are WISE (Slotta and Linn 2009) and Go-Lab.

## The Go-Lab ecosystem

The Go-Lab ecosystem was and is being developed in a set of EU-sponsored (FP7: Go-Lab, H2020 Next-Lab and GO-GA) and national projects. Its development began in 2013. The Go-Lab ecosystem consists of two main online platforms. The sharing and support platform (Golabz) presents all digital resources^2^ and is the main landing place, and the authoring and learning platform (Graasp) enables the co-creation and implementation of ILSs.

The Go-Lab ecosystem is distinct (unique) in the sense that it not only offers under a single umbrella the possibility to access and use publicly available interactive resources supporting STEM education, but also enables their creation or co-creation with pedagogical templates and their implementation with colleagues and students, as well as ultimately, if desired, their public sharing under creative commons licenses with the whole STEM community. The creation, the collaboration required for such creation, the personalization, and the sharing of digital resources are not common practices for the great majority of teachers. This paper discusses how the use of this complete ecosystem influences the instructional design practices of teachers, as well as their creation and sharing practices (see the section on the research questions for more details).

The Go-Lab ecosystem has been developed following the participatory design, responsive design, and privacy–by–design methodologies. The public resources are accessible without registration, that is, no personal data are collected. Teachers are in full control of their resource creation and the data collection in the ILSs they offer to their students. Students do not need to register to use ILSs. Thanks to this design and dedicated features, the ecosystem is fully compliant with the European General Data Protection Regulation (GDPR).

### Sharing and support platform (Golabz)

**Fig. 1:**
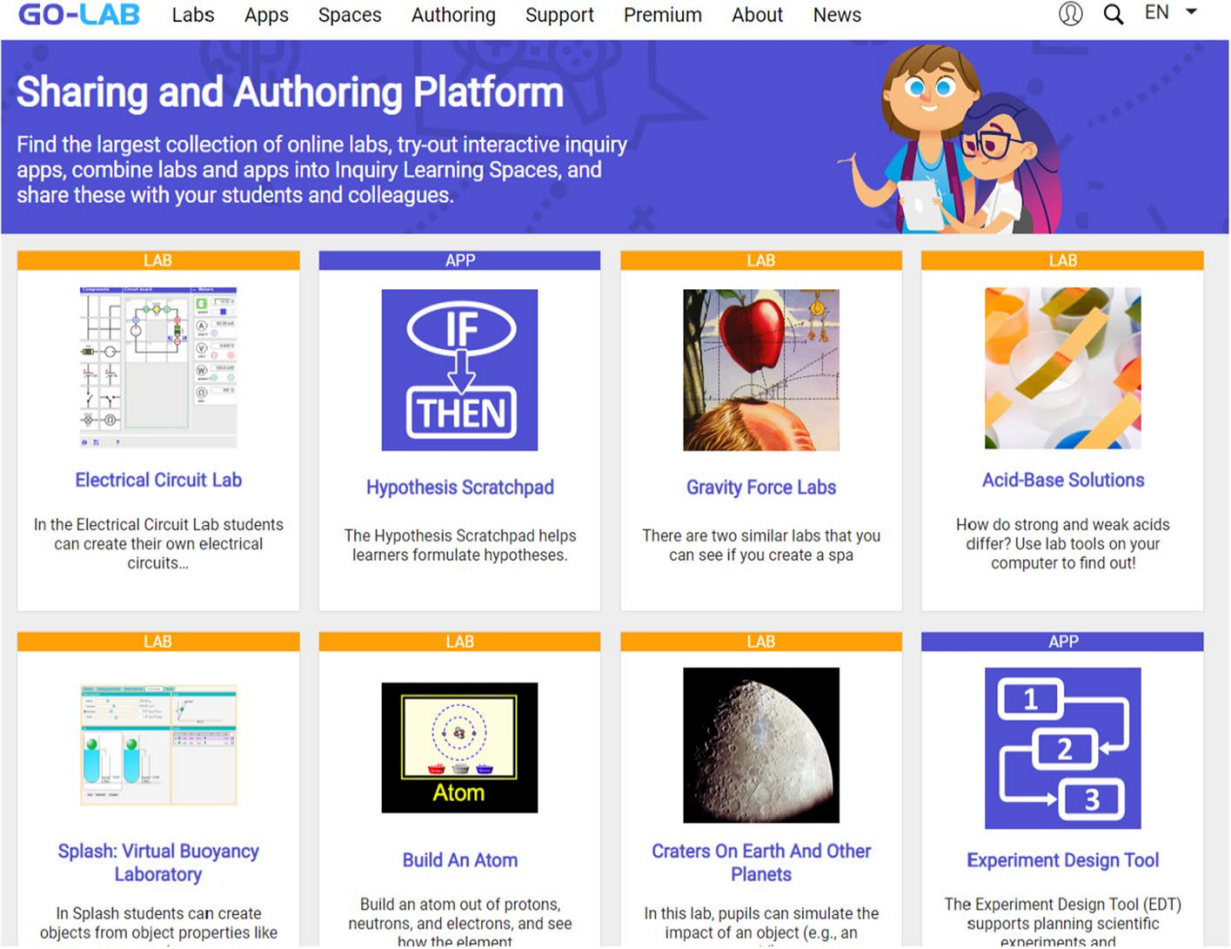
The Golabz homepage (www.golabz.eu)

The principal way the Go-Lab ecosystem is presented to a user is through the Go-Lab sharing and support platform. The homepage for the Go-lab sharing and support platform, called Golabz for short, is www.golabz.eu. On this platform, users (who are mainly teachers) can find: (a) online labs; (b) learning apps; (c) inquiry learning spaces, (d) a link to the Go-Lab authoring and learning platform (Graasp); (e) access to Go-Lab support (e.g., movies explaining the apps); (f) a link to the Go-Lab premium site, offering paid services and products; (g) an ‘about’ page with information about the projects that are associated with the ecosystem; and finally, (h) the Go-Lab news section. Figure 1 shows the Golabz landing page.

#### Online labs

At the end of 2019, Golabz held 614 labs, 54 of which were remote labs, 543 were virtual labs, and 17 referred to a data set. The majority of these labs were in the domain of physics, with 375 labs; there were 97 chemistry labs, 72 for mathematics, 62 for biology, 38 for astronomy, 35 for technology, 37 for environmental education, and 38 for engineering. These numbers add up to more than 614 because a lab can cover more than one content domain (e.g., physics and chemistry). At a deeper level, the online labs are labeled using more specific domain–related terms; for example, for physics, more specific classifications are electricity and magnetism (61 labs), energy (74 labs), fields, (22 labs), forces and motion (174 labs), and so forth. Teachers can find a lab by using a layered filtering system (including the domain, big ideas of science, lab type, student age, or language) or by searching using keywords.

#### Apps

Effective inquiry learning is not a fully student–directed enterprise, but seeks a balance between student freedom and guidance and support. This guidance can originate from different sources, for example, peer students, the teacher and, when available, a digital learning environment (e.g., Go-Lab ILSs). In these ILSs, one way to provide students with guidance and support is through apps, small digital tools that scaffold the student during inquiry processes, for example, hypothesis construction or experiment design. These apps are related to another student support mechanism, the Go-Lab inquiry cycle. This cycle (Pedaste et al. 2015) provides students with a series of steps or phases in the inquiry process, including orientation, conceptualization, investigation, conclusion, and discussion. These steps appear in an ILS as a series of tabs that students can enter. At the moment, a total of more than 40 apps are offered. The majority of these apps relate to learning processes in the Go-Lab inquiry cycle and can be included in an ILS to support the students in their inquiry process. Other apps enable additional learning and instructional processes such as testing of knowledge or teacher feedback, or refer to class management, such as an app that shows the teacher the phase of an ILS each student is in at a certain point in time.

#### Inquiry learning spaces (ILSs)

**Fig. 2:**
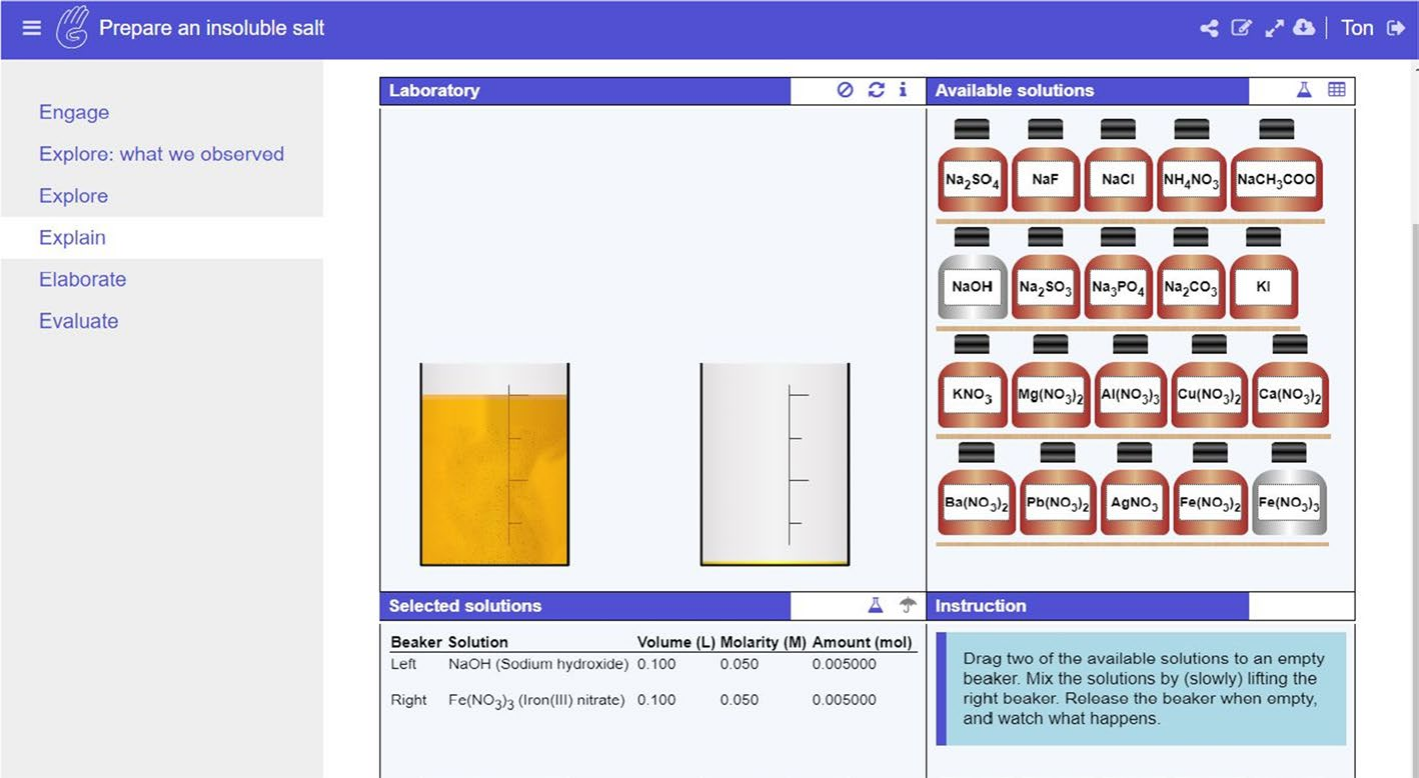
Example of an Inquiry learning Space (ILS)

Inquiry learning spaces are the online or digital spaces where students interact with the learning material, which may include apps, labs and other multimedia resources (mainly text, images, and videos). Such learning material is organized in a sequence of phases, with the Go-Lab inquiry cycle (Pedaste et al. 2015) as the default. The phases can be adapted by the teacher/designer (see later under the Go-Lab authoring and learning platform), and typically the lab is offered in the investigation phase. Figure 2 gives an example of an ILS on the physics topic of buoyancy; the Go-Lab inquiry cycle is depicted to the left and students can navigate through the cycle by clicking on a phase. Teachers can use their ILSs privately, but they can also offer them for publication on Golabz. By the end of 2020, teachers had shared close to 1100 ILSs in Golabz, so that other teachers can re-use and possibly adapt them.

**Fig. 3:**
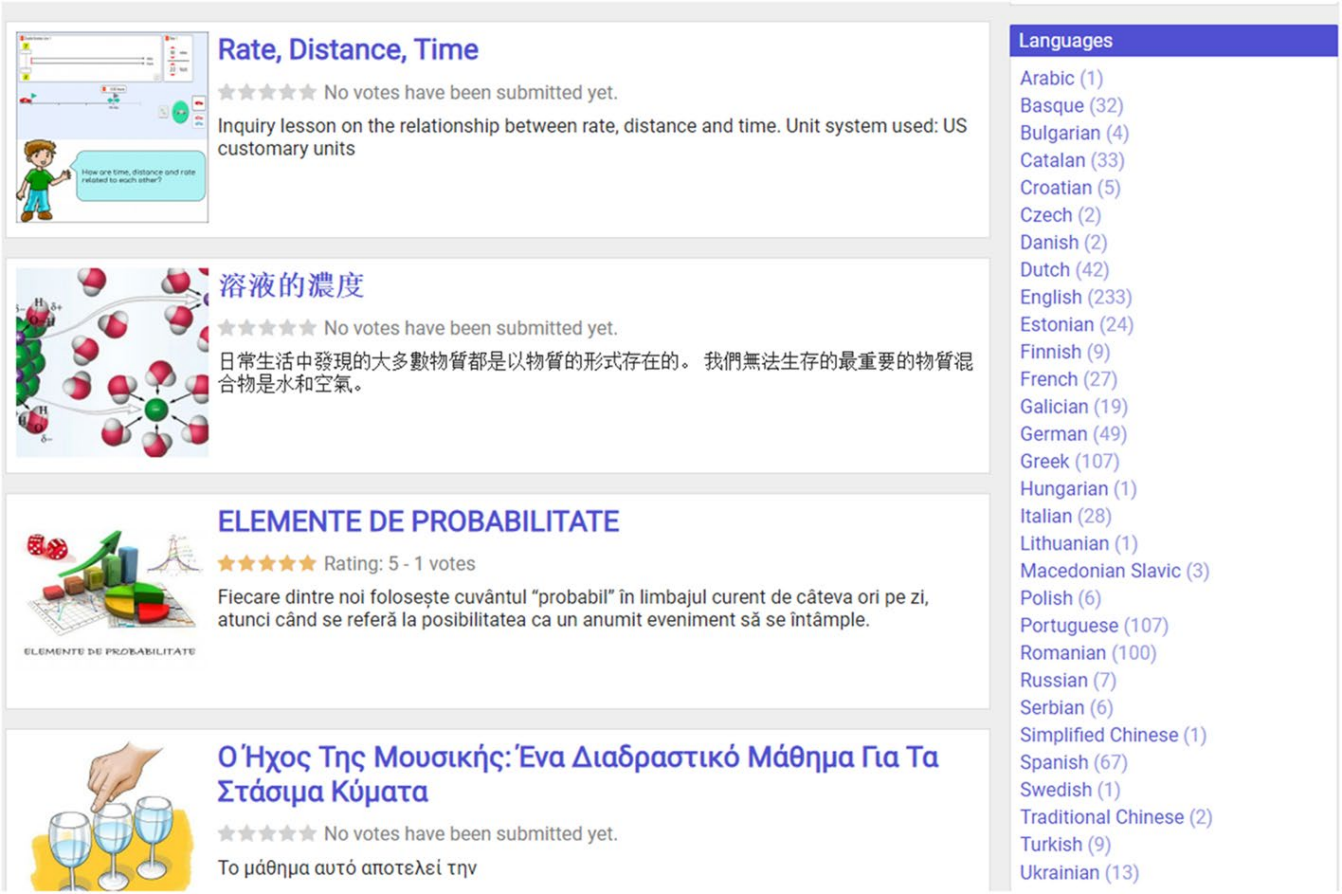
Languages used for published ILSs

The Go-Lab ecosystem has been translated into a multitude of languages, and ILSs are also created and offered in many different languages. Figure 3 lists all 30 languages used for ILSs that have been published on Golabz.

The major application domain for ILSs is physics. This makes sense, because most labs are from the physics domain, with biology second, and chemistry third. The great majority of ILSs are intended for students from 13 to 14 years and 15 to 16 years old, with each category containing close to 600 ILSs; some ILSs are classified as for more than one age level. Fewer ILSs are available for younger ages, as would be expected given that Go-Lab was initially developed for students from 12 to 18 years old, with apps specifically for younger age groups being created only recently.

### Authoring and learning platform (Graasp)

The design component of Go-Lab is based on Graasp, which is a general–purpose online platform supporting personal, collaborative, and inquiry learning, as well as knowledge sharing (Gillet et al. 2016, 2017). It was extended to serve as the Go-Lab authoring platform, enabling seamless (co)creation or personalization of inquiry learning spaces. Graasp (which is available at www.graasp.eu) also manages the runtime activities of an ILS, hence the term “learning” in the name of the platform. Both the Go-Lab sharing and support platform and the ILSs can be used without registration; Graasp, however, requires registration to enable teachers to create and access their resources.

There are three ways to create an ILS in Graasp. The first is when a teacher has found an interesting lab in Golabz. The teacher can click the “create space” button on the Golabz description of the lab, and is then automatically redirected to Graasp, where a personal version of this space is created and automatically populated with the phases from the Go-Lab inquiry cycle, with the selected lab in the Investigation phase. The second way to create an ILS is to start directly in Graasp and create an ILS from an available inquiry learning scenario, and then include the lab(s), apps, and multimedia material. The third way is to start from an existing ILS and adapt it. The existing ILS can be one that the same author created previously, an ILS that was published on Golabz by another teacher, or an ILS created by another teacher and shared in Graasp.

**Fig. 4:**
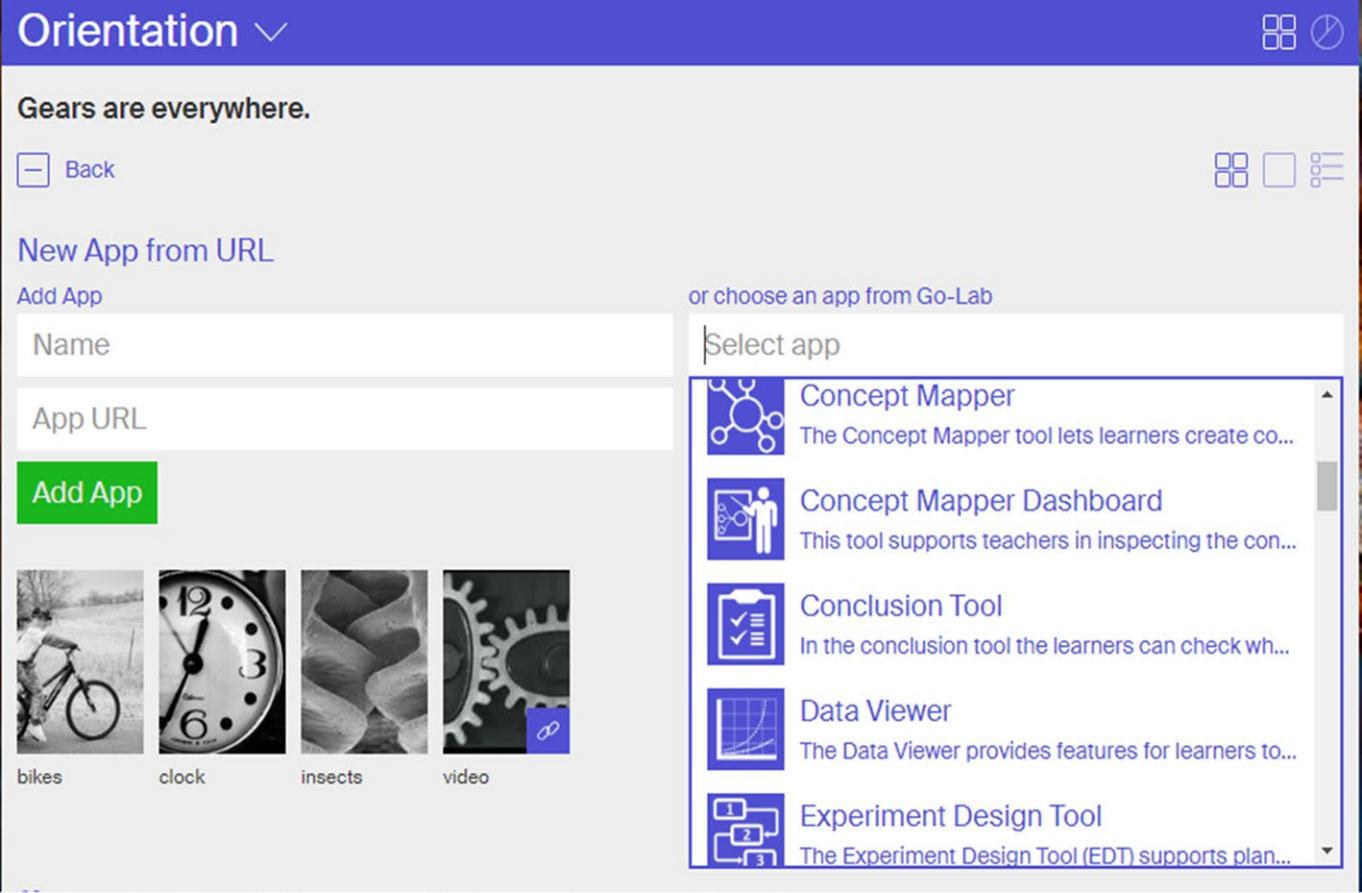
Adding an app to an ILS

When an ILS is created, the standard set of phases for a Go-Lab inquiry cycle are included, but the teacher can also adapt the number and names of the phases. After that, the teacher can start populating the inquiry cycle phases with labs, apps and multimedia material. As an example, Fig. 4 shows how a teacher can add an app by choosing from the list of available apps.

**Fig. 5:**
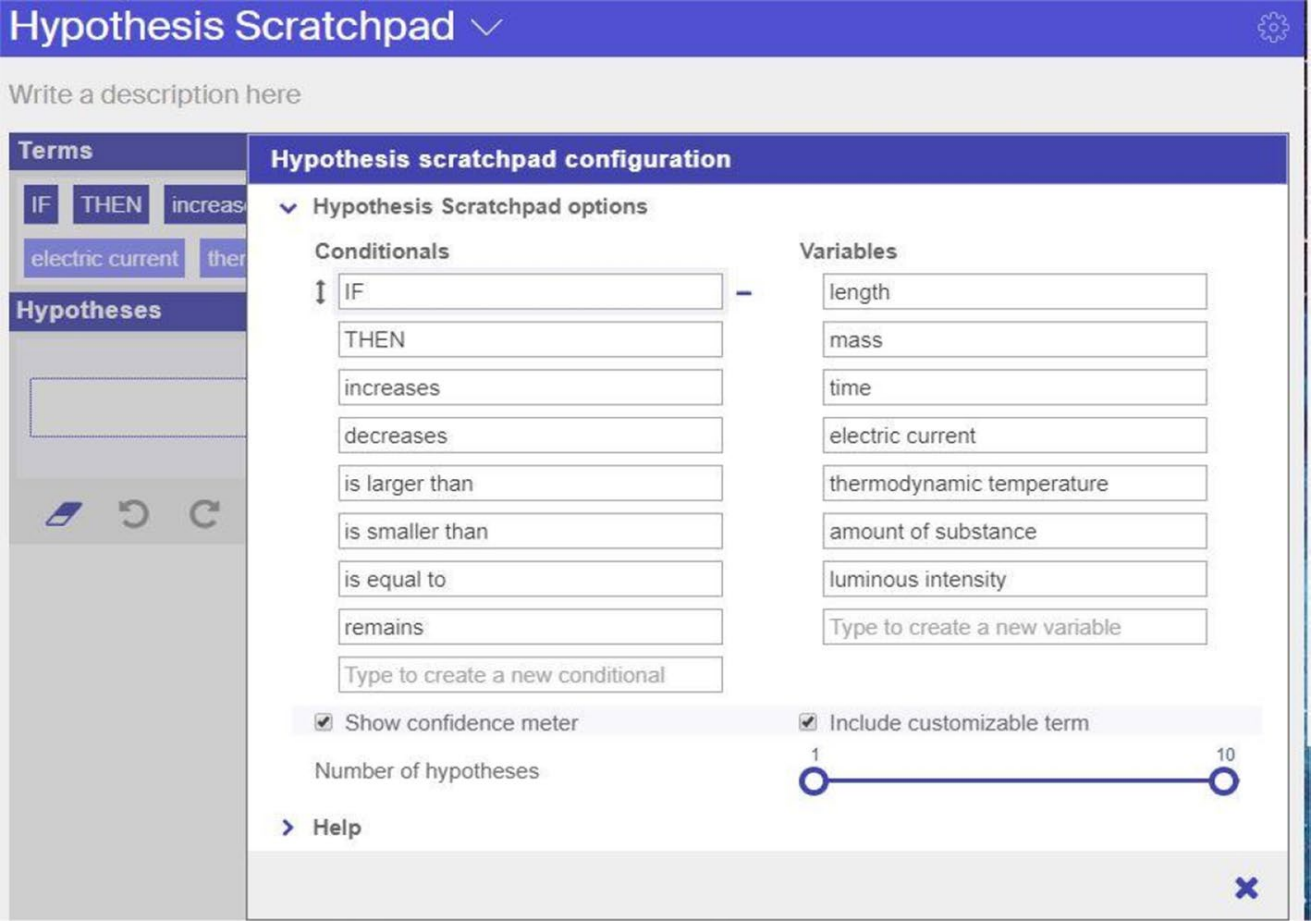
The app configuration process illustrated for the hypothesis scratchpad

Once the app is included in an ILS, the teacher can configure it, which means setting specific parameters for this app and inserting default content. Figure 5 shows the configuration of the hypothesis scratchpad as an example. The parameters that can be set are: the maximum number of hypotheses a student can enter, whether the ‘confidence meter’ will be shown or not, and whether students have the option to include terms of their own in the hypotheses they create or must use only the default terms. These default terms (conditionals and variables) can be determined by the teacher. In addition, the teacher can include half–hypotheses or ready–made hypotheses that students can use.

The Go-Lab authoring and learning platform has many more features, such as an optional student tracking mechanism for learning analytics and a discussion tool for communicating with other teachers. For further explanations and videos, see the Golabz support pages (https://support.golabz.eu/). A specific feature that is of relevance for the current paper is collaborative authoring. In Graasp, teachers can share an ILS and become co-authors. This means all teachers in the team can edit the ILS, inspect the history of the changes, and communicate through a chat.

After the personalization or creation of an ILS has been completed, teachers can share the ILS with their students by providing them with the URL of the ILS or by distributing a short URL or a QR code. Students do not need to register to use an ILS. In Graasp, the teacher can determine whether students can use the ILS anonymously or need to log in with a nickname or a nickname plus password. Teachers can also share their ILSs with the larger world by publishing the ILS on the Golabz sharing platform, so that other teachers can re-use these ILSs, possibly after having first adapted them. To publish, teachers need to complete a form with metadata (e.g., length of the ILS, subject domain, language, etc.). Next, the ILS is judged by the Go-Lab team on a number of basic quality criteria, and then it is published, after which it can be used as is, or copied and adapted by other teachers.

### Teacher support

As can be gauged from the above, the Go-Lab ecosystem provides different forms of support to the teacher/designer, with a current emphasis on the design phase. First, as mentioned earlier, teachers are offered all elements (labs and apps) needed for designing ILSs. Second, the design process is supported by providing teachers with an overall pedagogical structure (scenario) in which teachers can assemble the lab(s) and apps offered through Go-Lab, and complete it with other multimedia material. A dedicated set of scenarios can be used for structuring the ILS and shaping the students’ learning process, going from the “basic scenario”, which represents the “standard” inquiry cycle, to more advanced ones, involving collaboration between students, for instance. Third, while populating the basic scenario in the Go-Lab authoring and learning platform, teachers can ask for automatic suggestions as to which apps to use in the different phases of the ILS. Fourth, the sharing and support platform (Golabz) offers a larger set of design guidelines (tips & tricks) for more specific design decisions. Fifth, teachers can use ILSs completed by other teachers and published on Golabz as the starting point for their own designs.

Apart from all of the facilities outlined above, teachers are supported in their design process by courses they can complete (with a technical emphasis on using the ecosystem, more pedagogically oriented courses on inquiry–based learning, and combinations of both). These courses vary from a few hours long to multi-day workshops, and are offered as local initiatives at many places and in many languages. In addition, Golabz houses an extensive support page, with online videos, instructions, a MOOC, and pedagogical tips. An online support system gives teachers the opportunity to ask for specific support from the Go-Lab team.

### Adoption of the ecosystem

Over the lifespan of the ecosystem thus far (Go-Lab came online October 1st, 2014 and we report here data through December 31st, 2019), 442,146 different individuals visited the Go-Lab sharing and support platform, with a bounce rate of 47%. At the end of 2019, there were 37,683 registered users^3^ of the Go-Lab ecosystem, meaning that these individuals had created an account on the Go-Lab authoring and learning platform.^4^ This means roughly that close to 9% of the visitors to Golabz created an account on Graasp. When looking at these figures, it is important to take into account that the sharing and support platform can be visited in order to find an individual lab, without intending to create an ILS.

**Fig. 6:**
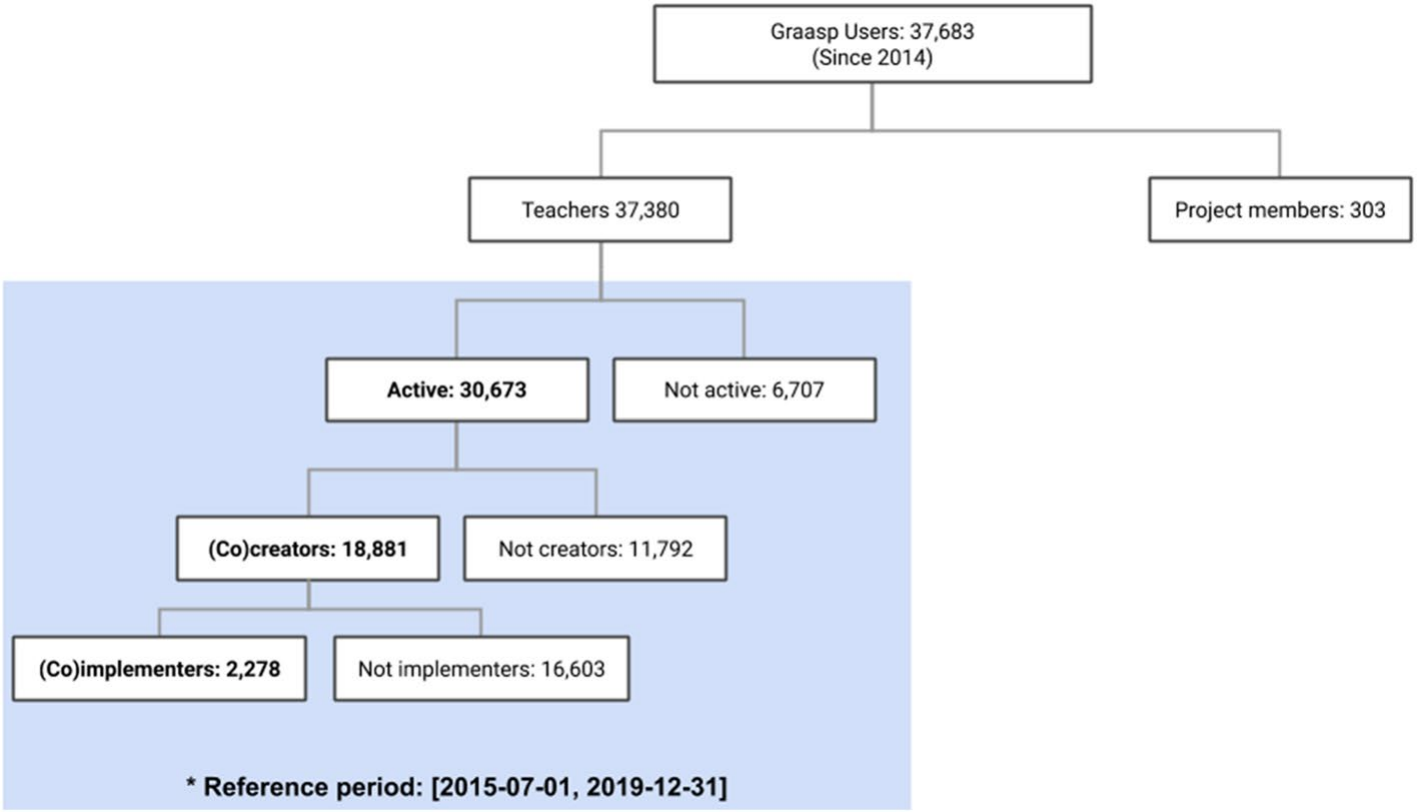
Overview of Graasp users, including the different levels of usage among teachers

Figure 6 offers an overview of Go-Lab users and their involvement with the platform. While 303 users are members of the projects that created the ecosystem, the rest are considered potential teachers. Since July 2015, when the platform started tracking user activity, 30,673 teachers have been active users (i.e., have used the platform). Among these active teachers, 18,881 were involved in the creation of an ILS and 2278 (potentially) implemented^5^ an ILS in the classroom, reaching 103,776 students. Although project members have also created and implemented ILSs by themselves without teacher involvement, this paper focuses on the ILSs implemented when teachers were involved.

Although not all teachers identified themselves as from primary, secondary or higher education, 3457 provided this information in their profile (around 11% of the active teachers). According to these profiles, 29.71% of the teachers come from primary education, 63.47% are from secondary and 16.78% come from higher education. It should be noted that teachers can be teaching primary and secondary, or secondary and higher education students at the same time, which means that there could be overlap between these groups.

The great majority of visitors to Golabz (39.07%) come from the United States, followed by Spain (5.47%), Romania (4.17%), India (3.40%), the United Kingdom (3.04%), Canada (2.96%), Italy (2.47%), the Netherlands (1.94%), Portugal (1.93%), and Greece (1.86%), among the top 10 countries. Interestingly, according to Graasp figures, the top countries of origin of the teachers implementing ILSs in the classroom differ: Switzerland leads (13.04%), followed by Estonia (11.33%), Portugal (10.76%), Spain (7.46%), the Netherlands (6.89%), France (5.71%), Ukraine (3.69%), Greece (3.64%), Germany (3.12%) and Finland (3.03%). Except for Ukraine, there are Go-Lab expertise centers in the rest of these countries that support the local community of users, which may explain why these countries have produced many implementers. The fact that teachers from all over the world (covering 49 different countries) use the ecosystem and become implementers despite lacking expertise centers also implies that teachers discover Go-Lab in different ways and that they are able to use the ecosystem with different levels of support.

## Research questions on the instructional design process

In the course of developing Go-Lab, a series of experimental studies aiming at the student level have been conducted, mainly to measure the effect of specific versions of apps (see e.g., van Riesen et al. 2018; Efstathiou et al. 2018; Kuang et al. 2020). The results of these studies were used for the (re)design of those Go-Lab apps. Studies at the teacher level have often been of a more qualitative nature and focused on gathering teachers’ ideas about requirements for the ecosystem or testing of interface designs (see e.g., Law et al. 2017). Now, with Go-Lab having been used by a large number of teachers over an extended period, together with the online registration of their actions, we have the opportunity to explore a set of instructional design issues with a large data set, which involves in–service teachers who used the ecosystem to design ILSs for actual classroom usage. The instructional design issues to be explored are related to the starting point for the design (empty templates or more concrete learning objects), the virtues and dynamics of the collaborative design process, and the objective of the design, where teachers decide if they want to use the ILS in the classroom and/or share it with their colleagues by publishing it on the Go-Lab sharing platform.

The first design issue concerns the starting point for a Go-Lab instructional design. Instructional design is a creative, but still structured process for which several ‘normative’ models exist, such as the classical ADDIE (see e.g., Branch 2009) and Dick and Carry (see, e.g., Dick 1996) models. These models are content free and basically offer a set of stages that designers of instructional material should go through in order to create instructional material. In Go-Lab, the instructional design process is supported not so much by offering design stages, but by offering ready–made, but adaptable and configurable, templates that teachers can fill with content. Usage of templates is seen as a way to increase the efficiency of the instructional design process (Roytek 2010), but it can also be a way to increase the quality of the resulting instructional material, in case a teacher or designer lacks the necessary pedagogical knowledge or needs support to translate abstract theoretical concepts into practical material (Yanchar et al. 2010). In Go-Lab, teachers are offered templates when designing ILSs, in the form of an overall inquiry cycle and (potentially partly configured) inquiry apps that focus on specific student inquiry activities (such as hypothesis creation). In their design, teachers need to fill in these templates and/or adapt the existing configuration. An alternative way of working is not to start from the pedagogical structure, but to start from the domain content, as is advocated in approaches that emphasize the use of learning objects (see, e.g., Wiley 2000). In Go-Lab, teachers can start designing an ILS from one of the labs in the lab repository at Golabz or they can use an existing ILS, also from Golabz, for example, one from another teacher (or their own) as the starting point for their design. When an existing ILS is used, an integration of domain and pedagogical structure is offered as a starting point, as is the case with learning objects. An analysis of the design process in Go-Lab gave us the opportunity to explore whether teachers preferred a design process that starts from empty pedagogical structures or a process that takes content as the starting point.

A second major (and growing) theme in instructional design is collaborative (participatory) design. Instructional designs require multiple forms of expertise, content knowledge, pedagogical knowledge, and in the case of designing digital material, technological expertise (Mishra and Koehler 2006). Grouping teachers or teachers with design and/or technological experts increases the chance that all needed expertise is available, and it may create an atmosphere of mutual improvement and progress (see, e.g., Cober et al. 2015; Martinez-Maldonado et al. 2017). In addition, designing in mixed teams of teachers and experts is seen as an excellent learning opportunity for teachers (see, e.g., Lee and Kim 2014; Voogt et al. 2015). Collaboration between individuals is facilitated by digital authoring systems. In our case, the authoring part of the Go-Lab ecosystem (Graasp) allows for online collaboration, in the sense that more than one person can design and create an ILS, changes made by different individuals can be monitored, and designers can exchange messages through a chat. Our second exploration, therefore, concerned the way collaboration between teachers and between teachers and project members occurred during the design process and how this affected the products that were designed (ILSs). In terms of design teams, we distinguished between individual teachers designing an ILS, one or more teachers who formed a team with a Go-Lab expert, and one or more teachers who collaborated in creating an ILS without a Go-Lab expert being present. We were interested to see how different forms of collaboration would influence the design process and the characteristics of the product that was designed (see, e.g., Brown et al. 2013).

A third exploration concerned the sharing of material by teachers with their students and with (international) colleagues. After having completed an ILS, the first decision teachers must make is whether they will offer this self–created digital material to their students. We were interested to see if we could see trends, over the years, in the confidence and willingness of teachers to use their own materials in the classroom, how long it took before a created ILSs was actually used, and the numbers of students involved in this. A second decision teachers must make is whether they want to share their work with others, nationally and even internationally. Studies have investigated the professional characteristics of teachers related to their inclination to use published and open digital material (Vermeulen et al. 2012) and the conditions that are necessary to realize this usage (Wills and Pegler 2016; Huber and Helm 2020). However, there is still very little information about the other side of the process, the willingness of teachers to make their own learning products public and to share their material with others. In Go-Lab, the technical hurdle to clear in order to publish material is very low. After having created an ILS, teachers can click a button, and after filling in a form with (straightforward) metadata, they can offer their ILS for publication (before actual publication a basic quality check is performed). However, there could be many other issues, for example, more psychological factors, that would prevent teachers from publishing their work, possibly in combination with the lack of a reward for publishing the material (Liber 2005). With the Go-Lab data we gathered, we could explore how publishing an ILS is related to structural aspects of the ILS and characteristics of the design process.

## Method

**Fig. 7:**
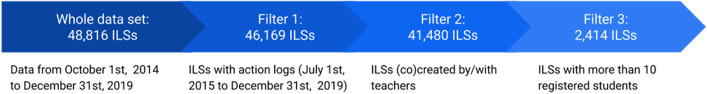
Filters applied in selecting ILSs for the dataset

The explorations described above were based on the traces and products generated in Graasp.^6^ To provide a solid dataset, several filters were used to select the relevant ILSs for our study (see Fig. 7). First of all, the period of analysis spans from July 2015, when user actions started to be systematically logged, up to and including December 2019. In this paper, we will call this period (July 1st, 2015–December 31st, 2019) our *reference period*. Second, since we are interested in tracing teacher practices, only those ILSs with teachers involved in their creation were considered in the analysis (i.e., ILSs created exclusively by project members were discarded). Third, some assumptions were also made regarding implementations. An ILS was considered implemented in the classroom when more than 10 students logged on to it (this empirical rule of thumb stems from conversations with teachers using the platform). We assume that including only ILSs with more than 10 different students logged on filtered out tryout ILSs and ILS in initial development. After taking these considerations into account, out of the 46,169 ILSs created during the reference period, 2414 ILSs (co)created by teachers and potentially implemented in the classroom were selected as our dataset.

We started the analysis of the dataset by identifying the composition of the design team, using the following (co)creation categories: single teacher; group of teachers; teacher(s) and project member(s). These categories reflect whether teachers worked individually or collaborated with either their peers or project members. Then, we analyzed several parameters per (co)creation category, for instance: design process characteristics (iterations, reuse of ILSs, number of students logged on), design effort (number of authors, design time, design actions), and structural parameters as to how ILSs were populated (number of phases, Golabz apps and labs, other apps/labs, and external resources such as links, images, videos, etc.). Because our data were not normally distributed, we used non-parametric statistics to examine main trends in the data; specifically, we used likelihood ratio chi–square tests for nominal data, Mann–Whitney and Kruskal–Wallis tests for comparing between two or three (co)creation categories, respectively, and Spearman’s rho indices for correlations between parameters. We also examined several parameters focused on ILS usage, including the number of created vs implemented ILSs, the number of students reached, and the number of published vs not published ILSs.

## Results

This section organizes the results around two main topics: how the authoring process took place and how the structure and content of the ILSs were designed. We have also included some findings regarding how the ILSs have been used.

### Design process characteristics

Although the starting point was not traceable for a small number of ILSs in the dataset (3.23%), the analysis did reveal the different paths followed by teachers while designing and creating an ILS. Out of the 2414 implemented ILSs, around 20% started from the Golabz sharing platform (13.46% were based on published ILSs and 7.76% were based on a published lab). This means that the majority (75.56%) of the implemented ILSs originated in Graasp, starting from an empty ILS that the teacher populated with materials. These figures show that while teachers often used the material available in the portal (published ILSs or labs) as a starting point to create their ILSs, they more often started from the structure provided by an empty ILS and populated that with materials available at Golabz (e.g., the Go-Lab apps and labs), external material (e.g., YouTube videos), and self–created content (e.g., instructional texts).

In Graasp, teachers can keep working on the same ILS until they implement and/or publish it; alternatively, they can make a copy of their ILS at some point and continue working on that copy until implementation and/or publication. They can also repeat that latter process and make multiple copies along the way. We have labelled copying and continuing to work as an *iteration*. When a teacher starts working on a published and/or implemented ILS, then makes a copy and continues working on that copy, the first step would then not count as an iteration, while the second (and following) steps would count as such. Longer chains of implementing, publishing, and iterating could also occur. Two-thirds (1636) of the implemented ILSs were developed from scratch, without iterations in the development process. The other one-third (778) of the implemented ILSs were the result of an iterative process, where teachers adapted previously published or implemented ILSs and/or progressively refined ILSs created from scratch until they were ready for implementation.

Our data set does not allow us to see the reasons behind moving from one version of an ILS to the next and we also cannot see whether teachers tried out versions of ILSs with their students before moving to a new version, as is the primary advice in classical instructional design models (see e.g., Branch 2009). Our clear impression, though, is that teachers often used a rapid prototyping approach that is supported by the facilities that Go-Lab offers, in which intermediate versions of the ILS form a basis for self–reflection or discussions with colleagues in order to take the next step (Tripp and Bichelmeyer 1990). Another reason to create a copy can be that once an ILS has been successfully implemented, teachers may create a new copy and share it, sometimes in an adapted form, with a new group of students, to keep the data from different classes separate.

### Design team composition

**Fig. 8:**
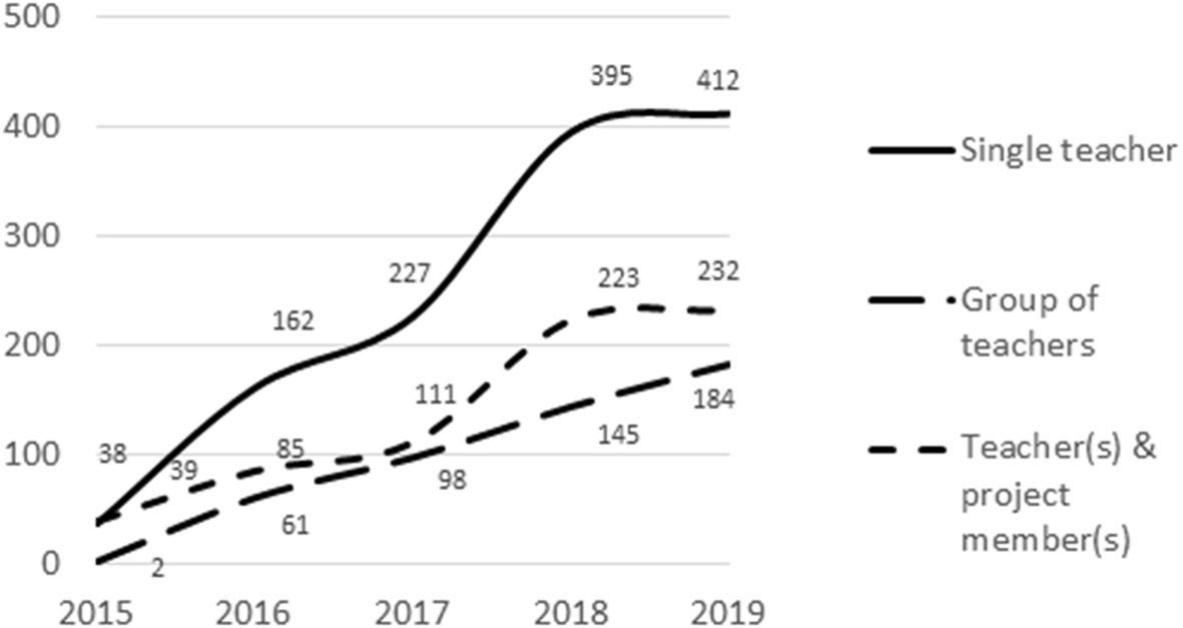
Number of implemented ILSs per (co)creation category and year

There was an even split among the implemented ILSs between ILSs created individually or collaboratively: 51.12% of the implemented ILSs were created by a single teacher and 48.33% were created as a team effort, with about 20.30% of the ILSs involving teams of only teachers and 28.58% involving project members as well.^7^ Looking at how the (co)creation of implemented ILSs evolved over the years (see Fig. 8), we can observe an interesting trend: there has been a sharper increase in the number of implemented ILSs created individually compared to the other two categories. This may be because ILSs were initially often created by teachers in some relation to the project (they participated in workshops with other teachers or worked together with project members), whereas over the course of time, teachers who were involved in the first stages of the project started to develop ILSs on their own and teachers (from all over the world) who had no relation whatsoever with the project team also started to develop ILSs on their own.

Table 1 presents design process characteristics per (co)creation category. These data reveal that implemented ILSs co-created by teachers and project members were more likely to have been iteratively designed, with intermediate versions; they also had a markedly higher chance of being reused later on than ILSs created by a single teacher or groups of teachers. These differences for ILSs co-created by teachers and project members indicate that they were subjected to a relatively more intensive process of refinement before reaching their final form and being implemented, and that they were also reused more after their implementation. The mean number of logged-on students was relatively higher for ILSs created only by teachers, either individually or in groups, as compared to ILSs created with the aid of project members [Mann–Whitney *Z* = −2.05, *p* < .05, for the difference between “single teacher” and “teacher(s) & project member(s)”; Mann–Whitney *Z* = −2.84, *p* < .01, for the difference between “group of teachers” and “teacher(s) & project member(s)”]. This difference in mean numbers of logged-on students may reflect that the implementation of ILSs created by teachers in collaboration with project members was sometimes in a preparatory phase, while the version of the ILS used in the classroom in the end was finalized by an individual teacher, potentially after having copied it.

**Table 1:** Design process characteristics per ILS (co)creation category for implemented ILSs

	Single teacher (*n* = 1234 ILSs)	Group of teachers (*n* = 490 ILSs)	Teacher(s) & project member(s) (*n* = 690 ILSs)	Statistic
Iterative design (%)	7.2	6.5	14.3	Likelihood ratio Chi–square = 29.85***
Reused later on (%)	20.3	30.4	49.0	Likelihood ratio Chi–square = 167.46***
Logged-on students (average number)	43.33	40.74	37.53	Kruskal–Wallis *H* = 8.59*

Note: * *p* < .05; *** *p* < .001

To assess the design efforts, we looked at three main aspects: the number of authors involved in the design process, the number of actions, and design time in Graasp. In addition, we analyzed whether individual authors devoted at least 10 min to designing. This threshold was determined by looking at the distribution of time invested per author. This distribution presented a clear gap at around 10 min, between very short bursts of actions lasting a few minutes (which probably did not enable thorough design effort) and a long tail of larger time investment. Looking at Table 2, if we compare the median number of authors participating in an ILS (first row) and the median number of authors who devoted at least 10 min (fourth row), we can observe a clear decrease for the two (co)creation categories that involved collaboration, either between peer teachers or between teachers and project members. Authors who devoted at least 10 min to designing were the ones who carried out the vast majority of the design actions (comparing the third and sixth rows in Table 2). For example, if we take the implemented ILSs created by teachers in cooperation with team members, there were 4 authors (on average), but only 2 were active for > 10 min, and these two carried out 1338.97 of the 1398.93 average actions. It is noteworthy that the total design time was below 10 min for 22.49% of the ILSs. While this amount of time could seem initially too low to generate an ILS, some of these ILSs were not created from scratch, but reused existing or published ILSs (44.94%), or at least labs available in the repository (8.29%). Some teachers also reported that they collected all of the material and the text to be integrated in an ILS in advance, thereby using the online creation time for just editing (copy–paste actions).

**Table 2:** Average or median values for design effort per ILS (co)creation category for implemented ILSs

	Single teacher (*n* = 1234 ILSs)	Group of teachers (*n* = 490 ILSs)	Teacher(s) & project member(s) (*n* = 690 ILSs)	Kruskal–Wallis *H*
Authors (median)	1	3	4	–
Design time (average min)	251	329	433	122.72***
Design actions (average number)	977	1125	1399	124.14***
Active authors (design time > 10 min; median)	1	1.5	2	–
Design time (average min for active authors > 10 min)	248	323	430	108.72***
Design actions (average number for active authors > 10 min)	936	1085	1339	103.05***

Note: *** *p* < .001

Design effort parameters (number of authors actively involved in design; design time; design actions) were closely correlated. The number of authors actively involved (min = 1; max = 41; median = 1) was correlated with the design time (Spearman’s rho = .48, *p* < .001) and the number of design actions (Spearman’s rho = .50, *p* < .001). Similarly, design time was also correlated with number of design actions (Spearman’s rho = .95, *p* < .001). As would be expected, when computing the same correlations only for authors who were active for more than 10 min, which would reflect the time needed to populate an ILS with basic applications and a virtual lab to design a complete inquiry cycle, significant coefficients were even higher: design time and number of actions again increased with the number of active authors (in both cases, Spearman’s rho = .69, *p* < .001), and design time increased with number of design actions (Spearman’s rho = .96, *p* < .001).

The values for all design parameters were significantly higher in the case of collaboration, especially when teachers collaborated with project members (Table 2). Collaboration between teachers increased design time significantly over the average time needed by individual teachers (Mann–Whitney *Z* = −4.79, *p* < .001; and Mann–Whitney *Z* = −4.39, *p* < .001 for design time when only active authors were taken into account), and the same trend was found for design actions (Mann–Whitney *Z* = −3.71, *p* < .001, for the difference between single teachers and groups of teachers; Mann–Whitney *Z* = −3.30, *p* < .01, for design time when only active authors were taken into account). Teachers who collaborated took more actions to complete their ILS design, as a group, than teachers working alone (Mann–Whitney *Z* = −3.25, *p* < .01; and Mann–Whitney *Z* = −2.75, *p* < .01 for number of actions when only active authors were taken into account). The values presented for teachers working together with project members are even higher across all variables in Table 2 when compared with the values for collaboration between peer teachers (for design time by active users: Mann–Whitney *Z* = −4.54, *p* < .001; for number of design actions by active users: Mann–Whitney *Z* = −5.42, *p* < .001). All of these differences indicate that collaboration increased design efforts significantly, especially collaboration between teachers and project members.

We also examined how team composition was related to structural aspects of implemented ILSs. Structural parameters related to phases showed that ILSs co-created by teachers and project members had more phases (see Table 3). On average, the ILSs had five phases (as in the 5-phase sequence proposed by the project). Nevertheless, authors adapted the sequence according to their needs; they removed phases (in 21.13% of the implemented ILSs) or added new ones (37.53%). The “conclusion” and “discussion” phases were more likely to be the phases that were removed (they were not included in 21.67% and 32.93% of the ILSs with removed phases, respectively). Based on our sampling of the ILSs that included extra phases, that decision was made mainly when some phases were too long and teachers split them into two, or when additional support material was provided to the students. Teachers could also decide to use a different inquiry cycle with different phases for their LS (see, for example, Fig. 2). With regard to adaptation of the default template in the implemented ILSs, the title of a phase was also often customized to make it understandable to the students (89.64%) and phases were populated with textual explanations (79.49%) and multimedia items (99.96%).

**Table 3:** Average values for structural parameters per ILS (co)creation category for implemented ILSs

	Single teacher (*n* = 1234 ILSs)	Group of teachers (*n* = 490 ILSs)	Teacher(s) & project member(s) (*n* = 690 ILSs)	Kruskal–Wallis *H*
Phases	5.21	5.49	5.65	43.51***
Golabz apps	7.24	7.71	11.37	131.14***
Golabz labs	.59	.60	.65	.20 ns
Other apps/labs	.04	.03	.07	6.14 ns
Resources	11.41	13.29	14.39	49.48***

Note: *ns*  non-significant; *** *p* < .001

For structural parameters related to how implemented ILSs were populated, ILSs created collaboratively also showed higher average values for number apps and labs, especially in the case of ILSs co-created by teachers and project members. An exception to this trend was the number of Golabz labs and apps or labs not available at the sharing platform, which did not vary significantly across categories. Mann–Whitney tests revealed that the number of Golabz apps increased significantly when ILSs were created collaboratively and especially when they were co-created with the aid of experts. The usage of external resources (links, images, videos, audios, documents, etc.) was also higher for ILSs created collaboratively than individually.

As far as the usage of Go-Lab apps and labs versus those from a third party, implemented ILSs used an average of 8–12 apps and close to 1 lab available in the ecosystem. The fact that some ILSs (3.02%) used external apps may be partly explained by an issue raised by some teachers: It was not realistic for the available apps and labs to cover the breadth of the curriculum and the language needs of all students. Thus, in some cases, it was necessary to find and embed external ad-hoc apps and labs.

### ILS usage: from authoring to implementation, sharing, and publishing

#### Created vs implemented ILSs

**Fig. 9:**
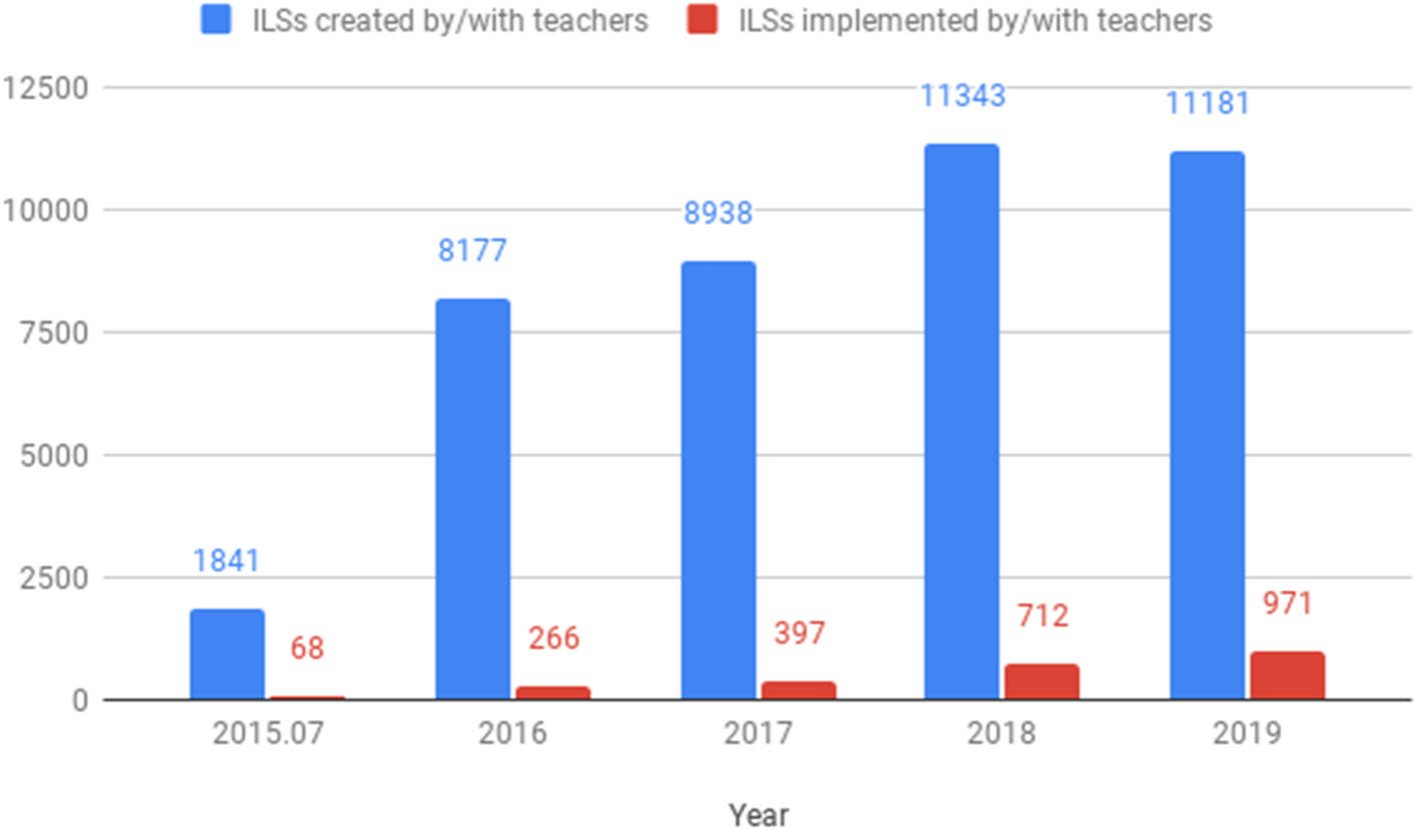
Numbers of ILSs created and implemented per year

During the reference period, a total of 18,881 teachers were involved in the creation of 41,480 ILSs. Figure 9 shows the distribution of created and implemented ILSs during the reference period, with a special note that data for 2015 started in July of that year. As can be seen, the number of created and implemented ILSs increased over time. While the proportion of implemented ILSs increased yearly, reaching 8.70% by the end of 2019, overall, an average of 5.8% of created ILSs were implemented over these years.

**Fig. 10:**
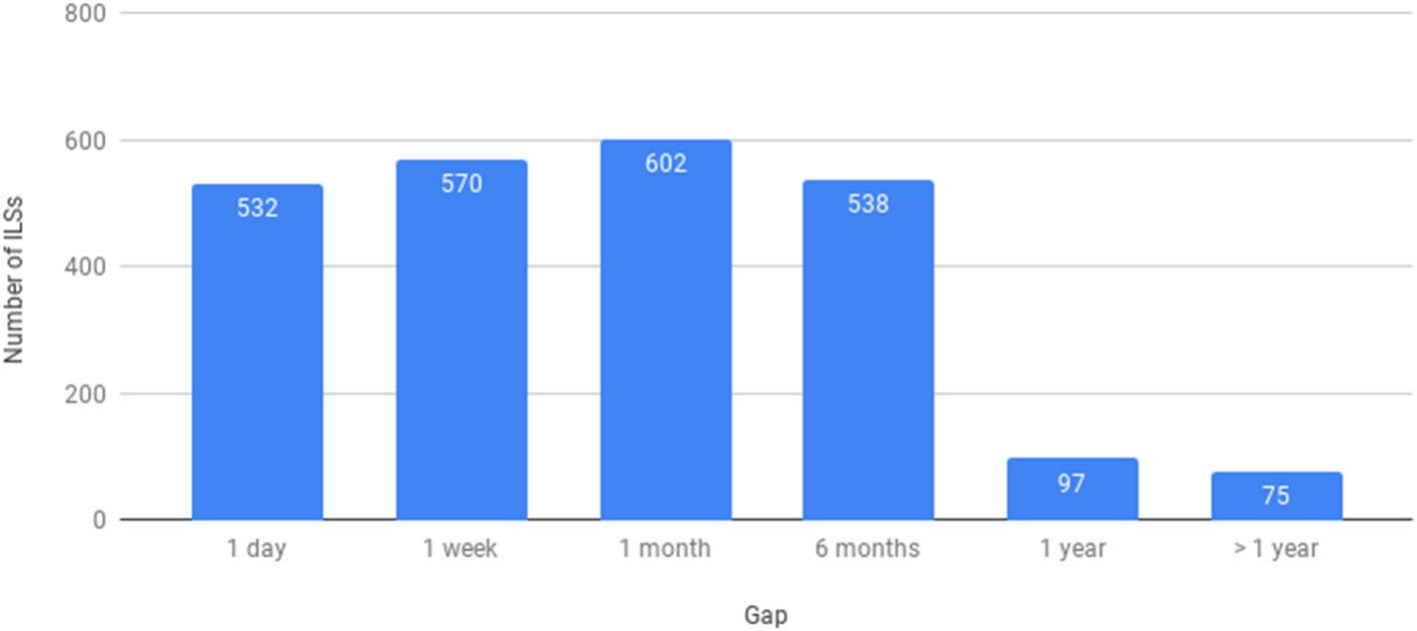
Time spent between the creation of an ILS and its implementation

The large gap between the number of ILSs created and the number implemented can most likely be explained by the fact that ILSs are also created as a trial to explore functionalities. A second explanation may be that, as depicted in Fig. 10, it often takes quite some time for a teacher to actually implement an ILS that has been created. In the dynamics of the classroom, many factors may lead to a delay, and then the next opportunity may be some months or even a year later.

#### Students reached

**Fig. 11:**
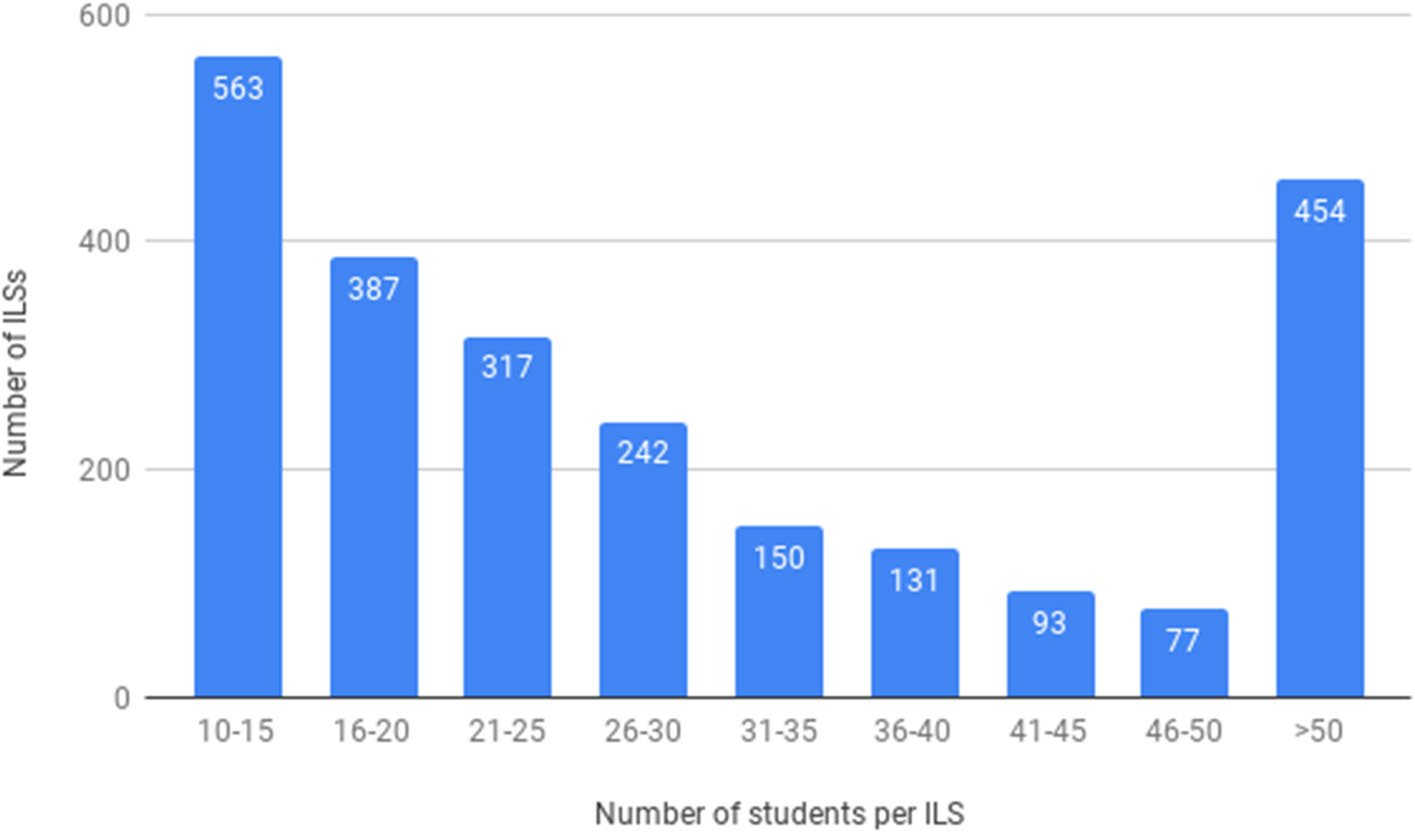
Distribution of implemented ILSs by number of logged-on students

A total of 99,321 students logged on to the 2414 implemented ILSs. Figure 11 depicts the number of logged-on students per implemented ILS. Many ILSs were used by 10–25 students, which most probably was one class (see Leuven and Oosterbeek 2018). As we have learned from our long–term contact with teachers, while sometimes students work individually, in some classes they work collaboratively using the same ILS while sitting in front of the computer together (e.g., depending on the students’ readiness for the tasks or the number of computers available in the room). Many ILSs show use by 25–50 or even more students, which is in line with our conjecture that ILSs are used in multiple classes.

#### Publication of ILSs

Out of 2414 implemented ILSs, 349 were published (14.46%). Table 4 displays a number of characteristic differences between published and unpublished ILSs. First of all, the design time spent on published ILSs was significantly higher than the time used for designing unpublished ILSs. A logistic regression (forward stepwise method; percentage of correctly predicted cases = 89.1%) showed that the odds of publishing an ILS increased with the number of times an ILS was reused (change in −2 log–likelihood = 352.47, *p* < .001), design time (aggregate time spent by users who remained active for more than 10 min; change in −2 log–likelihood = 6.51, *p* < .05), number of Golabz apps (change in −2 log–likelihood = 9.77, *p* < .01), and number of Golabz labs (change in −2 log–likelihood = 9.49, *p* < .01). In terms of outreach, published ILSs were copied more often than unpublished ones, as would be expected due to the higher visibility that the sharing platform offers, and they had more logged-on students; this latter difference was not significant. What may be more revealing is that published ILSs had more Golabz apps and resources. With regard to the design team, teacher(s) working with project member(s) were more likely to publish their ILS (25.7%) compared to a group of teachers (11.0%) or a single teacher (11.1%; likelihood ratio chi–square = 66.11, *p* < .001).

**Table 4:** Characteristics of published ILSs (mean values)

	Published ILSs (*n* = 349)	Unpublished ILSs (*n* = 2065)	Mann–Whitney *Z*
Design time (min)	463.97	294.30	−8.95***
Design time (min; active authors > 10 min)	457.49	288.68	−8.95***
Number of copies	10.93	.90	−30.40***
Number of logged-on students	50.98	39.48	−.94 ns
Number of Golabz apps	11.88	7.94	−10.98***
Number of resources	14.60	12.31	Z = −4.62***

Note: *ns* non-significant; *** *p* < .001

## Summary and conclusions

Until now, research on instructional design has generally been based on qualitative research with a small number of participants in authentic or experimental settings, or, when experimental studies are done or when more participants are involved, with student designers as participants. To our knowledge, this is the first time a large set of user data over an extensive period has been used to get a view of an instructional design process carried out in vivo, mainly by in-service teachers, for the design of (online or digital) learning material that is actually used in a classroom.

Designing learning environments that evoke students’ initial interest in the learning material, that keep them committed to the learning process, and that lead to (deep) conceptual knowledge is the ultimate aim of each and every instructional designer. The Go-Lab ecosystem was created to give teachers the facilities to use or create these types of learning environments, which in the case of Go-Lab are called Inquiry Learning Spaces (ILSs). In the current paper, we report an analysis of teachers’ use of the Go-Lab ecosystem over a period of 4.5 years. This data collection started roughly one year after Go-Lab became available online in its very first version. In the analysis, we focused on characteristics of the process of designing ILSs and the structure of the resulting ILSs. This paper, therefore, provides an overview of the different paths followed during the design process in the Go-Lab ecosystem (e.g., in terms of how to start the design process, with whom, and how to exploit its results), illustrating how the ecosystem supports the decisions made by teachers.

Since Go-Lab became available online, more than 440,000 individuals have visited the Go-Lab sharing and support platform (Golabz) and over 37,000 persons have registered on the Go-Lab authoring and learning platform (Graasp), which is required for authoring and using an ILS. This means that roughly 9% of Golabz visitors have created a Graasp registration, illustrating that Golabz can also be used for purposes other than ultimately creating and implementing an ILS. For example, Golabz offers much information on inquiry learning and is a one–stop shopping portal for a very large collection of online labs that can also be used in the educational process, which does not involve authoring and using an ILS. More than 80% of the persons who created an account were actually active in Graasp, and of these active users, over 60% (co)created an ILS. Finally, considering logging on by more than 10 students as the implementation criterion, 12% of the (co)creators of an ILS implemented the ILS in a classroom. We are not aware of any such figures for educational (or more generic) authoring software, so we cannot judge whether these figures are favorable or not, but we see these usage numbers as very positive. Making the move to creating something yourself and then using it in the classroom is a big step, both psychologically and timewise. The transition from authoring to implementation is also a process that could take quite some time. Even if a teacher has completed designing an ILS, it needs to be ready on time to fit the topics scheduled in the course plan, which may be often challenging, especially for topics occurring only once per year in the curriculum, or when the curriculum changes. Thus, this may slow down the implementation process. In addition, the Go-Lab ecosystem has seen large improvements after its first online introduction, which could have hindered early adoption. In this context, it is important to note that the implementation figures increased drastically over the years, from 68 classroom implementations in 2015 to 971 in 2019.

Our data show that, perhaps contrary to what might be expected, teachers prefer to start from empty pedagogical structures instead of starting from ready–made ILSs or available labs. This may show that even when teachers have in mind the domain that they want to include in the ILS, they also have a more or less clear idea of the approach they would like to use, and later fill that in with domain content. It may also indicate that teachers have idiosyncratic ideas about what they would like to do, and they do not want to make the effort to change all kinds of elements in existing ILSs. Nevertheless, a previous conceptualization phase (when teachers think about the ILS topic or get inspiration from other ILSs) may have taken place, but without leaving traces in the system. Future research should shed more light on the conditions under which teachers may prefer more or less structure and guidance in initiating their design process.

Designers collaborating to develop instructional material has the advantage that individuals are able to support each other, they can learn from each other and they can use the acquired knowledge in their individual work later as well (Rodríguez-Triana et al. in press). In our data set, roughly half of the ILSs that were implemented in the classroom were developed by a single teacher. The number of ILSs developed by teams of teachers or teams of teachers and project members accounted for the other half of the implemented ILSs, with around 30% developed by teams of teachers and project members and around 20% by teams of teachers. Concerning design effort, we see major differences between ILSs designed by single teachers and by teams of teachers or teachers and project members. Our data show that for ILSs created by a single teacher, average design time was more than 4 h, for ILS that were created by teams of teachers, average design time was close to 5.5 h, and average design time added up to more than 7 h for ILSs designed by teachers and project members. This is, of course, only online design time; we have no data on the design time used offline. The average number of design actions was considerably higher when groups of teachers created an ILS compared to single teachers, and this number was even higher when project members collaborated with teachers in the design team. In line with the higher design effort, teams of teachers and project members also generated intermediate versions of the ILS more often than single teachers or groups of teachers, meaning that they spent longer on refining their ILSs along the way. In addition, ILSs co-created by teachers and project members were reused more often. However, the average number of students logging on to implemented ILSs was higher for those designed by single teachers or groups of teachers compared to ILS designed by teams of teachers and project members. Most probably, this reflects the initial character of the implementation of the ILSs designed by mixed groups of teachers and project members, with single teachers making the final decision on the design of the ILS. We also see that collaboration added to the richness of the ILSs created. ILSs created in teams versus ILS created by single teachers had more phases and included more Golabz apps and resources.

A final step in ILS design is to offer the ILS for publication on Golabz. Once it is published on Golabz, other users can copy the ILS and use it in their classes as is or adapt it to their own needs. Publishing an ILS can be done simply by clicking the Publish button in Graasp and filling in a set of metadata. Before publishing an ILS, the Go-Lab team checks each offered ILS on a number of basic quality criteria (such as configuration of apps, language setting, etc.). ILSs that were published had longer average design time and were richer in terms of apps involved.

To summarize very briefly, our overall conclusion is that designers of ILSs start from the structure provided by the ecosystem (e.g., the Go-Lab inquiry cycle,), but that quite often they are able to make adaptations when they feel it to be necessary. This is true, despite the fact that, at the start, a teacher using Go-Lab already faces many challenges to the adoption of educational technology. An implemented ILS as presented in this paper is the result of many adoption steps taken by teachers, including accepting the risk of relying on digital technologies and network infrastructures, accepting the change from traditional teaching to active learning practices, accepting the implementation of online labs and open educational resources, being willing to create personal learning material, and last but not least, being (potentially) willing to share their product with colleagues and teacher communities at large. A second general conclusion is that collaborative design, either between peer teachers or when it involves experts, has advantages in terms of the richness of the resulting ILSs.

The current analysis gives initial insight into how a rich online educational design environment is used in practice. This Go-Lab usage is very versatile: it covers many countries and languages, different levels of teaching and different capabilities of teacher/designers. Collecting these data in vivo and with data protection and privacy rules in place also means that we do not have a full view of all relevant variables involved. This clearly points to some limitations to the analyses presented here. Another major limitation is that the data we present cannot give a full view of the design features used by teachers, because Go-Lab is an evolving ecosystem. For the analysis, this means that the ILSs in our sample came from different periods in Go-Lab history, with changes in the user interface and functionalities, available apps and labs, as well as support material and training. Thus, the general Go-Lab experience varied greatly over the ILSs analyzed, as did our capabilities to track user activity. Still, the data presented could be seen as valuable insofar as they open the black box of technology adoption that is normally kept closed. In any case, Go-Lab seems to be an exception to the rule that innovative instructional designs are only used in well–defined, closed, circumstances (Spector 2014).

By the time we were revising this manuscript (November 2020), the second wave of the COVID-19 pandemic was under way. The first wave had already impacted more than 1.5 billion students globally, endangering the continuation of STEM inquiry and experimentation in traditional, physical classrooms. During the COVID-19 crisis, we have observed a marked increase in the use of resources in the Go-Lab ecosystem, which could prove influential for teachers in the transition to digital STEM classrooms. Although there are several online resources available for digital STEM education, teachers may lack a comprehensive solution, able to integrate scattered digital resources, screen and select them to address their pedagogical design and implementation needs, and integrate them in digital lesson plans. The Go-Lab ecosystem presents such a holistic but simplified and user–tailored approach able to decrease the time needed and challenges encountered by teachers in the transition to digital STEM classrooms. Apart from the current strengths of the Go-Lab ecosystem (collection of labs, apps, and ILSs offered in different languages and accompanied by meta–data; Graasp authoring tool; support page), there are two additional assets of great importance for teachers in order to address classroom management and adverse effects on the development of twenty-first century skills for their students during the COVID-19 era. First, the Learning Analytics applications in the Go-Lab platform can allow teachers to track student performance and navigation in ILSs and to enact online formative assessment. Second, several labs and apps can be configured to allow for student online synchronous collaboration. These two options should be prioritized in future research on the Go-Lab ecosystem.

## Footnotes

1 In Graasp, designing an ILS is called the authoring process; this immediately includes the creation of the ILS. The person performing these activities is termed the author.

2 Go-Lab was originally set up as an online platform. Currently, also offline, digital, ILSs, with restricted functionality, can be created, especially for use in places with poor internet connections.

3 While technically the platform only registers created accounts, and the same person could have several accounts using different email addresses, for the sake of simplicity, we will refer to people with accounts as users (either teachers or project members).

4 In March 2020, after the start of the coronavirus crisis, the usage numbers had close to doubled.

5 We regard an ILS as implemented when it has > 10 different student users, see also later.

6 Despite the large number of digital traces, it should be noted that our analysis leaves out the offline design steps, since there was no way (without overloading the teachers and potentially hindering their usage) to systematically collect these data from such a large number of users, the great majority of whom were unknown to the research team.

7 The design team composition was determined by taking a snapshot of the persons involved in an ILS at the end of 2019. This means that any changes in team composition during the process were not taken into account.
